# Editorial: Insights in livestock genomics

**DOI:** 10.3389/fgene.2026.1840925

**Published:** 2026-04-30

**Authors:** Xiao-Lin Wu, Shi-Yi Chen, Johann Sölkner, Marino Cassandro

**Affiliations:** 1 Council on Dairy Cattle Breeding, Bowie, MD, United States; 2 Department of Animal and Dairy Sciences, University of Wisconsin–Madison, Madison, WI, United States; 3 Farm Animal Germplasm Resources and Biotech Breeding Key Laboratory of Sichuan Province, Sichuan Agricultural University, Chengdu, China; 4 Department of Sustainable Agricultural Systems, Division of Livestock Sciences, University of Natural Resources and Life Sciences, Vienna, Austria; 5 National Association of Italian Holstein, Brown and Jersey Breeders, Cremona, Italy; 6 Department of Agronomy, Food, Natural Resources, Animals and Environment, University of Padova, Padova, Italy

**Keywords:** complex traits, domestic animals, epitranscriptome, genomics, GWAS, proteins, RNA

## Introduction

Livestock genomics has entered a transformative period in the third decade of the twenty-first century. The field has progressed decisively beyond low-density marker panels and traditional genome-wide association studies, embracing genomics, transcriptomics, epigenomics, epitranscriptomics, single-cell technologies, regulatory network modeling, and machine learning–enabled dissection of complex traits. The sixteen contributions assembled in the Research Topic “*Insights in Livestock Genomics*” collectively exemplify this transformation. These studies highlight not only technological maturation but also new insights into the genetic basis of production traits, environmental adaptation, disease resistance, and genomic diversity across a wide range of livestock and aquaculture species, including cattle, sheep, goats, pigs, buffalo, deer, and fish.

## Genomic foundations: architecture, diversity, and adaptation

A comprehensive understanding of livestock biology begins with robust genomic foundations. High-quality genome assemblies and population-scale resequencing efforts provide the structural and evolutionary context within which functional and quantitative analyses can be interpreted.

At the structural level, Zhu et al. presented a chromosome-level genome assembly of *Hypomesus nipponensis* using long-read sequencing technologies, markedly improving contiguity and completeness relative to earlier drafts. Their analysis reveals expansion of transposable elements and tandem repeats, accompanied by paralogous gene family expansion and chromosomal rearrangements during divergence. Although focused on a commercial fish species, these findings underscore that genome architecture is dynamic, with repetitive elements and structural variation actively shaping evolutionary trajectories. High-quality reference genomes clarify evolutionary relationships and provide essential frameworks for variant discovery and comparative genomics.

Beyond structural genomics, multiple contributions examine genetic diversity and selection signatures across populations. Gao et al. investigated fat-tailed and thin-tailed sheep through whole-genome resequencing, identifying candidate genes linked to lipid metabolism and tail fat deposition, thereby illuminating how selection has molded adaptive energy-storage phenotypes. Song et al. explored high-altitude adaptation in Tibetan sheep, detecting selection signals in pathways associated with hypoxia response, metabolism, and ultraviolet tolerance. Together, these studies demonstrate how genomic scans reveal molecular bases of environmental resilience relevant to both evolutionary biology and climate-adaptive breeding.

Genomic resequencing analyses also inform conservation and sustainable breeding. Pi et al. characterized Tahe red deer, reporting elevated inbreeding and reduced diversity alongside candidate genes associated with adaptation to arid environments, highlighting the balance between improvement and conservation. Zhan et al. showed that Tongjiang goats maintain relatively high diversity and low inbreeding while harboring unique selection signatures related to development, reproduction, and immune function, reinforcing the importance of preserving locally adapted genetic resources.

Similarly, Ramoroka et al. examined non-descript cattle in South African smallholder systems, revealing moderate to high diversity but substantial introgression from commercial breeds. Their findings illustrate the complexity of managing genetic resources in low-input environments, where informal crossbreeding may enhance productivity yet threaten breed distinctiveness. Genomic characterization in such systems provides a scientific basis for structured breeding programs that balance genetic gain with long-term sustainability.

These studies show that genomic foundations extend beyond sequence assembly to encompass diversity, population structure, and adaptive signatures. By integrating genomic data with ecological and production contexts, they establish the evolutionary landscape upon which functional and quantitative genomics can build.

## Regulatory and functional genomics: from single cells to epitranscriptomics

While population genomics reveals how variation is distributed and selected, understanding how it translates into phenotype requires mechanistic insight. A defining feature of this Research Topic is the emphasis on regulatory and functional genomics across biological scales—from single-cell resolution to epitranscriptomic and non-coding RNA regulation—illustrating a shift from variant cataloging to molecular dissection.

At the cellular level, Han et al. applied single-cell RNA sequencing to the regenerating antler tip of sika deer, generating a high-resolution atlas of cell populations involved in rapid elongation and ossification. Reconstructed differentiation trajectories and shared ligand–receptor interactions reveal coordinated cellular communication underlying one of the fastest examples of mammalian organ regeneration. Such approaches move beyond bulk transcriptomics to uncover cell-type–specific regulatory programs.

Epitranscriptomic regulation further expands this regulatory landscape. Liao et al. mapped N6-methyladenosine (m6A) modifications across porcine tissues using nanopore direct RNA sequencing, identifying tissue-specific methylation patterns and associations with transcript abundance and alternative splicing. These findings highlight RNA modification as an additional layer of phenotypic regulation beyond DNA sequence variation.

Several studies have dissected regulatory mechanisms influencing economically important traits and disease responses. Gao et al. characterized the transcription factor ELF5 in buffalo mammary epithelial cells, demonstrating its role in regulating milk protein synthesis via the JAK2–STAT5 and PI3K/AKT/mTOR pathways. By combining molecular analyses and functional assays, the study links transcriptional regulation directly to lactation biology.

Non-coding RNAs also emerge as central regulatory players. Badia-Bringué et al. identified lncRNAs associated with paratuberculosis infection in cattle, connecting differential expression patterns with immune-related genomic regions. Chen et al. integrated lncRNA and mRNA profiles across developmental stages in Ningxiang pigs, identifying co-expression modules and candidate genes involved in intramuscular fat deposition. These integrative analyses illustrate how non-coding transcripts participate in complex regulatory networks affecting both health and production traits.

Expanding further, Li et al. analyzed endogenous retrovirus (ERV)-derived transcripts across goat tissues, revealing developmental and infection-associated activation patterns. Co-expression between ERV reads and immune-related genes suggests that repetitive elements function as regulatory components rather than passive genomic remnants.

By integrating single-cell, epitranscriptomic, transcription factor, and non-coding RNA analyses, these studies deepen our understanding of how genomic information is interpreted within cells and tissues, providing mechanistic foundations for biologically informed breeding strategies.

## Genetic architecture of complex traits and emerging analytical approaches

Understanding how genomic variation translates into economically important phenotypes remains a central objective of livestock genomics. While regulatory studies elucidate biological mechanisms at the molecular level, quantitative genomics provides the statistical framework for connecting genome-wide variation to complex traits relevant to production, efficiency, and profitability. Several contributions in this Research Topic exemplify the continued refinement of trait dissection methodologies and the integration of advanced computational tools.

Baneh et al. investigated the genomic architecture of carcass and meat quality traits in Angus cattle through genomic variance partitioning using whole-genome sequence data. By decomposing genetic variance across minor allele frequency bins, functional annotation categories, and chromosomes, the study demonstrates that while intergenic and intronic variants contribute substantially to total genetic variance, per-SNP contributions are typically larger for genic variants, particularly those located in exonic regions. Importantly, joint multi-component modeling yields heritability estimates consistent with whole-genome analyses, underscoring the value of integrating multiple genomic relationship matrices. Such partitioning approaches enhance our understanding of how different classes of variants contribute to complex traits and inform strategies for genomic prediction and marker prioritization.

Genome-wide association studies (GWAS) remain a powerful tool for identifying candidate loci underlying production traits. Wang et al. conducted GWAS in Simmental cattle for milk production, body size, and tail-related characteristics, identifying genes associated with growth, morphology, and metabolic inflammation. Similarly, Ma et al. performed GWAS in a Landrace pig population for backfat thickness and feed conversion ratio, detecting multiple significant genomic regions and annotating candidate genes involved in growth and metabolic pathways. These studies reinforce the polygenic nature of commercially important traits while simultaneously highlighting specific loci that may serve as targets for marker-assisted or genomic selection.

Complementing classical GWAS approaches, Shi et al. applied machine learning methods to RNA-seq data to identify genes influencing intramuscular fat deposition in pigs. By combining support vector machine recursive feature elimination and random forest models, they narrowed a large set of differentially expressed genes to a small number of intersecting candidates enriched in lipid metabolism pathways. This approach illustrates how machine learning can enhance feature selection and improve signal detection in high-dimensional omics datasets. Rather than replacing statistical genetics, machine learning methods increasingly function as complementary tools that refine candidate gene prioritization and capture nonlinear patterns not easily modeled by traditional linear frameworks.

Together, these studies reflect the evolution of quantitative genomics toward integrative and computationally sophisticated models. As datasets expand in scale and complexity, analytical innovation will remain essential for translating genomic discovery into accurate prediction and effective selection.

## Challenges and future perspectives

Several challenges remain as livestock genomics moves toward greater integration and application. A primary challenge is bridging association and causality. While genomic scans and transcriptomic analyses increasingly identify candidate genes and pathways, functional validation and mechanistic confirmation remain essential for translating discoveries into robust breeding strategies. Integrating multi-omics layers at population scale—linking genome sequence, regulatory variation, and phenotypic performance—will be critical for improving predictive accuracy and biological interpretability. Despite rapid advances in livestock genomics, a substantial gap remains between research discoveries and their practical application in breeding programs and livestock production systems. The reduction of cost for single animals and the customize chip SNPs for specific traits of economic and social interest might be improve the diffusion and the reduction on the gap between theoretical and practical use.

Important areas not prominently represented in this Research Topic also warrant continued attention. These include pangenome and structural variant analysis, large-scale genomic prediction theory and optimal mating strategies, high-throughput phenomics, host–microbiome interactions, crossbreeding optimization using sexed semen combined with genomics predictions, and genomic approaches to climate resilience and environmental sustainability, just to list a few. Expanding efforts in these domains will further strengthen the connection between discovery and application.

Looking ahead, livestock genomics is evolving toward a more reliably predictive and design-oriented domain. By integrating high-resolution molecular insight with quantitative and computational advances, the field is increasingly positioned to deliver precision breeding strategies that enhance productivity while safeguarding genetic diversity and long-term sustainability.

